# Correlations between Perceived Oral Malodor Levels and Self-Reported Oral Complaints

**DOI:** 10.1155/2015/343527

**Published:** 2015-07-27

**Authors:** Atsushi Kameyama, Kurumi Ishii, Sachiyo Tomita, Chihiro Tatsuta, Toshiko Sugiyama, Yoichi Ishizuka, Toshiyuki Takahashi, Masatake Tsunoda

**Affiliations:** ^1^Department of Endodontics and Clinical Cariology, Tokyo Dental College, Tokyo, Japan; ^2^Division of General Dentistry, Tokyo Dental College Chiba Hospital, Chiba, Japan; ^3^Tokyo Dental College School of Dental Hygiene, Chiba, Japan; ^4^Department of Periodontology, Tokyo Dental College, Tokyo, Japan; ^5^Department of Epidemiology and Public Health, Tokyo Dental College, Tokyo, Japan

## Abstract

*Objectives*. Even though objective data indicating the absence of oral malodor are presented to patients, they may be skeptical about the results, possibly due to the presence of some discomfort in the oral cavity. The objective of this study was to investigate whether there is an association among self-perceptions of oral malodor, oral complaints, and the actual oral malodor test result. *Materials and Methods*. Questions concerning self-perceptions of oral malodor and subjective intraoral symptoms were extracted from a questionnaire on oral malodor completed by 363 subjects who visited the clinic for oral malodor of Tokyo Dental College Chiba Hospital and gave consent to this study. In addition, the association of self-perception of oral malodor with values obtained after organoleptic and OralChroma measurement was analyzed. *Results*. No correlation between 195 subjects (54%) who were judged “with oral malodor” (organoleptic score of ≥1) and 294 subjects (81.6%) who had a self-perceptions of oral malodor was observed. Self-perception of oral malodor was significantly correlated with tongue coating (*p* = 0.002) and a strange intraoral taste (*p* = 0.016). *Conclusions*. Subjects with a self-perception of oral malodor were not necessarily consistent with those actually having an oral malodor. In addition, it was suggested that patients became aware of oral malodor when they felt oral complaints.

## 1. Introduction 

Increasing awareness of cleanliness by society in general has heightened interest in odor. Since one cannot sense oral malodor accurately by oneself, bad breath brings marked psychological discomfort and may interfere with social interactions [1]. Many people feel anxiety or distress regarding oral malodor, even though they may have no such oral malodor. They may believe they have oral malodor when they see someone touching his/her nose or grimacing during a conversation [2].

Even though a dentist judges the oral malodor level by smelling and informs patients that there is no oral malodor, it is difficult for them to accept it because the procedure is based on the examiner's subjectivity. Therefore, it is necessary to measure the actual oral malodor level by measuring major oral malodor-related volatile sulfur compounds (VSCs) contained in exhaled air, hydrogen sulfide (H_2_S), methyl mercaptan (CH_3_SH), and dimethyl sulfide ((CH_3_)_2_S), and “objectively” inform patients of the actual oral malodor level [3]. Many pseudohalitosis patients are convinced and relieved when the examiner (dentists and dental hygienists) inform them that there is no need to worry about oral malodor by showing the VSC measurement results [3, 4].

On the other hand, some people visiting an outpatient clinic for oral malodor are skeptical even though these objective data are presented [5]. They may be diagnosed with “halitophobia” based on the classification reported by Murata et al. [6]. However, if they have some subjective intraoral symptoms (self-reported oral complaints), these may induce pseudohalitosis or real malodor. If so, their distress caused by oral malodor is not resolved unless oral complaints are resolved. Therefore, it is necessary to investigate whether there is an association between a self-perception of oral complaints and actual manifestation of oral malodor or number of complaints of pseudohalitosis.

To clarify the association between the presence or absence of oral complaints and actual oral malodor measurement results, it was necessary to investigate the association between various subjective intraoral symptoms and oral malodor parameters (VSCs) in subjects who initially visited the outpatient clinic for oral malodor of Tokyo Dental College Chiba Hospital. The following null hypotheses were set: (1) there is no correlation between self-perceptions of oral malodor and oral complaints and (2) there is no correlation between self-perception of oral malodor and oral malodor parameters.

## 2. Materials and Methods

### 2.1. Subjects

Of a total of 429 persons who visited the outpatient clinic for oral malodor at the Tokyo Dental College Chiba Hospital between January 2009 and December 2011, 363 (123 males and 240 females) gave written consent after an explanation of the objective of the study. Four of them were excluded because of data deficiency. This study was performed after approval by the Ethics Committee of Tokyo Dental College (protocol approval number 375).

### 2.2. Extraction of Responses to Questionnaire concerning Oral Malodor

All initial examinees completed to a self-administered questionnaire concerning oral malodor before examination of the oral cavity and the oral malodor test. Questions concerning the presence or absence of subjective oral malodor symptoms and related items (11 items in total) were extracted and adopted for this study as follows.

*Questionnaire on Oral Malodor Responded to by Examinees of the Outpatient Clinic for Oral Malodor of Tokyo Dental College Chiba Hospital (List of Factors concerning Oral Complaints)*.

1. Do you think you have bad breath?
   (Yes/No/Unsure)
2. Do your gums bleed during tooth brushing?
   (Yes/No)
3. Do your gums ooze out pus?
   (Yes/No)
4. Do you have a loose tooth/teeth?
   (Yes/No)
5. Do you grind your teeth while you are asleep?
   (Yes/No)
6. Does your mouth feel dry?
   (Yes/No)
7. Is your mouth viscous?
   (Yes/No)
8. Is your tongue frequently coated with deposits?
   (Yes/No)
9. Do you often remove tongue coating?
   (Yes/No)
10. Do you notice a bad taste in your mouth?
   (Yes/No)
11. Is there medicine you have to take regularly?
   (Yes/No)

### 2.3. Evaluation of Oral Malodor Level

The oral malodor level was objectively evaluated by measuring the levels of 3 volatile sulfur compounds (VSCs) contained in intraoral gas using a portable gas chromatograph, OralChroma (FIS, Itami, Hyogo, Japan), and performing an organoleptic test.

To gather optimal test results, several precautions should be taken before the examinations: the patient should refrain from eating spicy foods, garlic, or onions the day before the examination. For at least 12 h before the consultation, the teeth should not be cleaned or rinsed, perfumes should be avoided, and, at least 6 h before the examination, the intake of food and liquids should be avoided. Smoking should be refrained from for at least 24 h before any examination [7].

To use breath odor at waking as the standard, measurement was performed between 9:30–11:30 a.m. [8]. The organoleptic test was performed employing 5-step grading: scores 0 = absence of odor, 1 = barely appreciable odor, 2 = moderate malodor, 3 = strong malodor, and 4 = severe malodor.

Prior to measurement using OralChroma, the patients were instructed to close their mouth and breathe through their nose for 30 seconds. A 1-mL disposable syringe (Top, Tokyo, Japan) was placed in the mouth through the lips and teeth, 1 mL of air in the mouth was aspirated, and 0.5 mL of this was immediately injected into OralChroma [9]. It was judged that oral malodor can be perceived when the H_2_S level exceeds 600 ppb, the CH_3_SH level exceeds 100 ppb, (CH_3_)_2_S level exceeds 100 ppb [2, 10], or organoleptic level is 1–4.

### 2.4. Analysis

The associations between self-perception of oral malodor and each subjective intraoral symptom and oral malodor parameter were investigated using the Chi-square test (*p* = 0.05). When data involved only 20 or fewer subjects, Yates' correction was applied. The association between the parameters was analyzed using Spearman's rank-correlation coefficient test. Statistical analysis was performed using IBM SPSS statistics 18 for Windows (IBM Japan Inc., Tokyo, Japan).

## 3. Results

### 3.1. Correlation between Oral Malodor Parameters

The associations among the 4 oral malodor parameters are shown in Table 1. Significant correlations were noted between the parameters of all combinations (*p* < 0.001). Particularly, a strong correlation was noted between the organoleptic test result and CH_3_SH (*r* = 0.906, *p* < 0.001).

### 3.2. Association between Self-Perception of Oral Malodor and Oral Malodor Parameters

Only 9 (2.5%) of the 359 subjects responded that they were not aware of their own oral malodor, 294 subjects (81.9%) responded that they had self-perceived oral malodor, and 56 subjects (15.6%) stated that they did not know. The results of analysis of the association between self-perception of oral malodor and the results of VSC measurement using OralChroma and the organoleptic test are shown in Table 2. Subjects who chose “unsure” to the questions on self-perception of oral malodor were regarded as having no self-perception. No significant correlation with the presence or absence of self-perception of oral malodor was noted in any of H_2_S, CH_3_SH, (CH_3_)_2_S, or organoleptic test results.

### 3.3. Association between Self-Perception of Oral Malodor and Oral Complaints

The results of analysis of the association between self-perception of oral malodor and elements of oral complaints are shown in Table 3. No significant correlation was noted between the presence or absence of periodontal disease-associated intraoral symptoms and self-perception of oral malodor. In contrast, a significant correlation was noted between tongue coating and self-perception of oral malodor (*p* = 0.002). A significant correlation with a strange intraoral taste was also observed (*p* = 0.016), but no significant correlation with intraoral viscosity was noted (*p* = 0.067).

The combinations of significantly correlated factors on analysis using Spearman's correction are shown in Table 4. Significant correlations were noted between oral dryness and intraoral viscosity, a strange taste, tongue coating, and frequent tongue brushing (*p* < 0.05). In addition, significant correlations were noted between intraoral viscosity and tongue coating and a strange intraoral taste (*p* < 0.05).

## 4. Discussion 

The objective of this study was to clarify the association between the presence or absence of subjective intraoral symptoms and results of actual oral malodor measurement. The oral malodor level was judged based on the measurement of 3 VSCs, in addition to the organoleptic test. The organoleptic test is considered to be the gold-standard oral malodor test because whether oral malodor is perceived by human olfaction is important for the judgment of the presence or absence of oral malodor [11, 12]. However, tests employing human olfaction cannot be quantified because it is based on subjective judgment by the examiner (operator). Therefore, comparison of the test results between before and after treatment is difficult, and the test lacks persuasiveness as a material for explanation of the oral malodor level [3]. Therefore, to judge the oral malodor level, generally, the measurement of oral malodor-producing substances using an oral malodor-analyzing device is concomitantly employed in addition to the olfaction-based organoleptic test.

The organoleptic score is correlated with the levels of VSCs contained in intraoral gas determined using gas chromatography and a portable sulfide monitor. We also observed correlations among the 4 items: the H_2_S, CH_3_SH, and (CH_3_)_2_S levels and organoleptic test result (*p* < 0.001). Particularly, the correlation between the organoleptic test result and CH_3_SH level was markedly stronger than those between the organoleptic test result and H_2_S and (CH_3_)_2_S levels (*r* = 0.906, *p* < 0.001). (CH_3_)_2_S stimulates olfaction at 1/6 and 1/21,000 of the levels of H_2_S and ammonia, respectively [13]. Accordingly, the strength of oral malodor is strongly correlated with the (CH_3_)_2_S level [14].

Of the subjects who visited the outpatient clinic for oral malodor during the 3-year period, only 9 subjects (2.5%) felt that they had no oral malodor and more than 80% of the examinees felt that they had oral malodor. However, only 54.3% of them were judged as having some level of oral malodor on the organoleptic test, showing no correlation between their self-perception and the organoleptic test results (*r* = 0.080, *p* = 0.128). This finding suggests that persons who self-perceive oral malodor are not necessarily consistent with those who actually have it, and this finding was consistent with those of several reports [14, 15].

About 60% of the examinees felt oral dryness. A significant association was present between self-perception of oral malodor and oral dryness. Indeed, oral dryness due to reduced salivary flow is a cause of oral malodor [16], and the oral malodor level rises with a decrease in salivary flow [17]. On the other hand, feeling oral dryness was not significantly correlated in this study with the organoleptic test result, H_2_S, or CH_3_SH, suggesting that a feeling of oral dryness does not necessarily indicate the actual reduction of salivary flow [18].

A significant association was also noted between self-perceptions of oral malodor and frequent tongue coating. Tongue coating has been reported to be one of the typical factors of oral malodor [19, 20]. Amou et al. [21] visually evaluated the accumulation of tongue coating using Kojima's 5-step scoring criteria [22] and observed its correlations with the organoleptic score and CH_3_SH level. Particularly,* F. nucleatum *and* T. denticola* contained in tongue coating have been reported to be closely involved in VSC production [23, 24]. Therefore, tongue cleaning is recommended to improve oral malodor [25–28]. A significant correlation was noted between tongue coating and frequent tongue brushing (*r* = 0.355, *p* < 0.001), possibly because patients know that tongue coating removal improves oral malodor. This was also demonstrated by the findings that frequent tongue brushing was inversely correlated with the organoleptic test result and CH_3_SH level.

A feeling of oral dryness was also significantly correlated with subjective symptoms causing oral complaints, such as intraoral viscosity and a strange taste. Similarly, a significant correlation was noted between a feeling of oral dryness and tongue coating (*p* < 0.001). These findings suggest that persons who feel oral dryness tend to have some oral complaints, and tongue coating induced their anxiety regarding oral malodor. Based on these findings, first null hypotheses that there is no correlation between self-perception of oral malodor and oral complaints was rejected.

It was also determined that there is no association between self-perception of oral malodor and its actual presence. Thus, the second null hypothesis was accepted. This is because examinees/patients suspected having oral malodor even though it was not present when there was some oral complaints such as, for example, the feeling of oral dryness, tongue coating, and a strange intraoral taste. Particularly, when oral dryness is felt, the person becomes sensitive to intraoral viscosity and an unpleasant taste, aggravating the tendency. Pseudohalitosis patients, that is, persons who visit outpatient clinics for oral malodor although it is actually absent, do not necessarily self-perceive oral malodor simply due to mental stress. The study results therefore suggest that some forms of oral complaints induced self-perception of oral malodor. On interviews of halitosis patients in actual medical practice, it is necessary to sufficiently ask whether they have discomfort in the oral cavity, in addition to asking about anxiety and mental distress due to oral malodor. If this is the case, not only mental support but also instruction and treatment should be offered to improve such oral complaints.

## 5. Conclusions 

Our study revealed that the subjects with a self-perception of oral malodor were not necessarily consistent with those actually having an oral malodor. In addition, it was suggested that patients became aware of oral malodor when they felt oral complaints.

## Figures and Tables

**Table 1:** Correlation coefficients between the results of 4 parameters regarding oral malodor.

	*n*	Spearman's correlation		
		CH_3_SH	(CH_3_)_2_S	OT
H_2_S				
<600 ppb	335	*r* = 0.247	*r* = 0.449	*r* = 0.422
≥600 ppb	24	*p* < 0.001	*p* < 0.001	*p* < 0.001
CH_3_SH				
<100 ppb	165		*r* = 0.294	*r* = 0.906
≥100 ppb	194		*p* < 0.001	*p* < 0.001
(CH_3_)_2_S				
<100 ppb	305			*r* = 0.472
≥100 ppb	54			*p* < 0.001
OT				
Score 0	164			
Score 1	83			
Score 2	59			
Score 3	26			
Score 4	27			

OT: organoleptic test; *n*: number of subjects.

*r*: correlation coefficient.

**Table 2:** Relationships between self-perception of oral malodor and associated parameters.

	Self-perceived oral malodor		*p* value
	(+) (*n* = 294)	(−) (*n* = 65)	
H_2_S			
<600 ppb	22	2	0.311 NS
≥600 ppb	272	63	
CH_3_SH			
<100 ppb	153	41	0.106 NS
≥100 ppb	141	24	
(CH_3_)_2_S			
<100 ppb	46	8	0.624 NS
≥100 ppb	248	57	
Organoleptic test			
Score 0	140	24	0.117 NS
Score 1	63	20	
Score 2	49	10	
Score 3	18	8	
Score 4	24	3	

NS: no significant difference (Chi-square test).

**Table 3:** Relationships between self-perception of oral malodor and oral complaints.

	Self-perceived oral malodor		*p* value
	(+)	(−)	
Bleeding from gum			
Yes	83	19	0.872 NS
No	211	46	
Pus from gum			
Yes	27	2	0.167 NS
No	267	63	
Tooth movement			
Yes	29	9	0.471 NS
No	265	56	
Grinding of teeth			
Yes	58	14	0.742 NS
No	236	51	
Mouth dryness^*∗*^			
Yes	181	29	0.012
No	113	36	
Intraoral viscosity			
Yes	192	36	0.133 NS
No	102	29	
Tongue coating^*∗*^			
Yes	200	31	0.002
No	94	34	
Frequent tongue brushing			
Yes	181	32	0.067 NS
No	113	33	
Strange taste^*∗*^			
Yes	99	12	0.016
No	195	53	
Taking medicine			
Yes	141	27	0.348 NS
No	153	38	

^*∗*^Significant difference (Chi-square test; *p* < 0.05).

**Table 4:** Correlated parameters analyzed by Spearman's correlation.

		*r*	*p* value
Bleeding from gum	Pus from gum	0.153	0.004

Tooth movement	Grinding of teeth	0.122	0.021

Mouth dryness	Intraoral viscosity	0.289	<0.001
	Tongue coating	0.199	<0.001
	Strange taste	0.197	<0.001
	Frequent tongue cleaning	0.108	0.040

Intraoral viscosity	Tongue coating	0.269	<0.001
	Strange taste	0.232	<0.001

Tongue coating	Frequent tongue cleaning	0.355	<0.001
	Strange taste	0.259	<0.001
